# 12-month outcomes of GLP - 1 in severe pediatric obesity: real-world data

**DOI:** 10.3389/fendo.2025.1663499

**Published:** 2025-09-19

**Authors:** Louise Cominato, Mariana L. Resende, Natalia Bernardes, Ludmila L. Rachid, Caroline G. B. Passone, Larissa B. F. Mattar, Georgia Neme, Sarah G. Souza, Claudia Renata P. Santos, Ruth R. Franco, Durval Damiani

**Affiliations:** Department of Pediatric Endocrinology, Institute for Children and Adolescents, Hospital das Clínicas, Faculty of Medicine, University of Sao Paulo (USP), São Paulo, Brazil

**Keywords:** GLP - 1 analog, obesity, treatment, children, adolescents

## Abstract

**Background:**

Real-world data on liraglutide for pediatric obesity is limited, especially in public healthcare systems of low- and middle-income countries.

**Objective:**

To evaluate the real-world effectiveness and safety of liraglutide in managing severe obesity among children and adolescents with severe obesity treated at a public quaternary hospital in Brazil.

**Methods:**

This cohort study included patients aged 6–17.9 years with BMI Z-score (Z-BMI) ≥+3, treated with liraglutide (up to 3.0 mg/day) and lifestyle intervention. Outcomes included changes in BMI, Z-BMI, weight, WHtR, metabolic markers, and adverse events over 3 months, 6 months and 12 months.

**Results:**

Of 74 patients, 55 completed 6 months and 22 completed 12 months. In patients aged 6–12 years (n=23), median Z-BMI decreased from +3.90 (3.4 - 5.1) to +3.06 (2.7 - 3.7) (p < 0.0001), and WHtR from 0.70 (0.66 - 0.73) to 0.64 (0.60 - 0.68) (p < 0.0001); 82.6% achieved ≥5% BMI reduction and 47.8% ≥10% BMI reduction. In adolescents aged ≥12 years (n=32), Z-BMI declined from +3.77 (3.02 - 4.66) to +3.48 (2.64 - 4.34) (p < 0.0001), and WHtR from 0.74 (0.62 - 0.80) to 0.67 (0.58 - 0.76) (p < 0.0001); 73.9% achieved ≥5% and 43.5% ≥10% BMI reduction. Improvements were also observed in LDL cholesterol, HbA1c, and HOMA-IR. Adverse events were mild and transient.

**Conclusion:**

Liraglutide was effective and safe in reducing adiposity and improving metabolic health in children and adolescents with severe obesity in a real-world setting.

## Introduction

Childhood obesity is a strong predictor of obesity in adolescence and adulthood, with long-term implications for health and quality of life. In 2016, more than 340 million children and adolescents worldwide were overweight or living with obesity, with prevalence rates continuing to rise in both high- and low-to-middle–income countries (1, 2). According to the World Health Organization (WHO), obesity is defined as abnormal or excessive fat accumulation that presents a health risk. In children aged ≥5 years and adolescents, obesity is defined as a body mass index (BMI) greater than +2 standard deviations (SD), and severe obesity as greater than +3 SDs (1–3). This chronic, progressive disease is associated with multiple metabolic and cardiovascular complications, including type 2 diabetes mellitus (T2DM), metabolic dysfunction–associated steatotic liver disease (MASLD), and increased adult mortality due to cardiovascular disease (2, 4, 5).

Lifestyle interventions—comprising dietary modification, physical activity, and behavioral counseling—are the cornerstone of pediatric obesity management. However, even intensive behavioral programs generally result in modest and often unsustainable BMI reductions (3, 6, 7). Guidelines recommend such intensive approaches as first-line treatment, but their feasibility is limited in real-world settings due to the time, specialized resources, and family engagement required (3, 6, 7).

The optimal timing for initiating pharmacotherapy remains debated. Expert consensus suggests that anti-obesity medication (AOM) may be considered after approximately six months of unsuccessful lifestyle-based treatment, particularly when patient motivation declines (8). Despite evidence from randomized controlled trials showing that AOM can achieve BMI reductions, use in pediatric populations remains limited. This is due, in part, to stigma and misconceptions that frame obesity because of insufficient willpower, rather than as a complex, multifactorial, and biologically influenced disease (9–11). Such stigma—directed toward both children and their caregivers—can delay initiation of evidence-based therapies and reduce adherence, underscoring the importance of addressing societal attitudes as part of treatment strategies (9, 12, 13).

Glucagon-like peptide-1 receptor agonists (GLP - 1 RAs) have emerged as promising pharmacological options. These agents promote satiety, reduce appetite, delay gastric emptying, and improve glycemic control by enhancing glucose-dependent insulin secretion and reducing glucagon levels (6, 11, 12). Liraglutide and semaglutide are currently approved by the US Food and Drug Administration (FDA) and the Brazilian Health Regulatory Agency (ANVISA) for adolescents aged ≥12 years, as adjuncts to lifestyle interventions (8, 12, 14). Clinical trials have demonstrated their efficacy and safety in reducing BMI and improving cardiometabolic risk factors without significant neuropsychiatric or cardiovascular risks (4, 15–17).

Despite growing evidence, real-world data on GLP - 1 RA use in pediatric populations—particularly from non–industry-sponsored studies in public healthcare systems of low- and middle-income countries—remain scarce (13). This study evaluates the effectiveness and tolerability of liraglutide in adolescents with obesity treated in a Brazilian public tertiary care center, addressing a critical evidence gap.

## Objective

To evaluate the real-world efficacy and safety of liraglutide in children and adolescents with severe obesity treated in a university-affiliated public quaternary hospital in Brazil. The primary outcomes were changes in Body Mass Index (BMI), body mass index z-score (Z-BMI), weight, and waist-to-height ratio (WHtR). Secondary outcomes included lipid profile (LDL and HDL cholesterol, triglycerides), glycemic control parameters (HbA1c, HOMA-IR), inflammatory marker (C-reactive protein= CRP), uric acid levels. Safety outcomes comprised the incidence of adverse events and assessment of linear growth and hepatic enzymes.

## Methods

This is a cohort study conducted in a quaternary public referral center for pediatric endocrinology in Brazil. The study was approved by the institutional ethics committee (CAAE: 67589823.0.0000.0068), and all participants and/or legal guardians provided informed consent before initiation of liraglutide.

Eligible participants were children (6–11 years) and adolescents (12–19 years) diagnosed with severe obesity, defined as BMI-for-age > +3 SDS according to the WHO 2007 growth reference, who had received liraglutide therapy for at least one dose between January 2021 and February 2023. Patients with diabetes or secondary causes of obesity, such as endocrine or genetic syndromes, were excluded.

Liraglutide was prescribed off-label following national clinical protocols, starting at 0.6 mg/day and titrated up to 3.0 mg/day based on tolerance and clinical response. All patients received concurrent lifestyle counseling by a multidisciplinary team (nutritionist, psychologist, endocrinologist).

Demographic, anthropometric, and metabolic data were retrieved from electronic medical records at baseline, 3, 6, and 12 months. Demographic variables included age, sex and Tanner stage. Primary outcomes included changes in BMI z-score and waist circumference. Secondary outcomes included changes in BMI, height, weight, blood pressure, fasting glucose, HOMA-IR, CRP, lipid profile, and liver enzymes. HbA1c was measured by high-performance liquid chromatography (HPLC); HOMA-IR was calculated using the formula: fasting insulin (μU/mL) × fasting glucose (mg/dL)/405.Z-BMI was calculated using WHO AnthroPlus software.

Data are presented as means ± standard deviations for continuous variables and as percentages for categorical variables, as appropriate. Paired *t*-tests or Wilcoxon signed-rank tests were used to compare baseline (T0) and 6-month (T6) values and between T6 and 12 months extension period (T12), depending on normality. For repeated measures across multiple time points (T0, T3, T6), the Friedman test was applied for non-parametric longitudinal analysis. A two-tailed *p* < 0.05 was considered statistically significant. All analyses were performed using GraphPad Prism Version 10.5.0.

Adherence was evaluated at each follow-up by structured patient self-report regarding missed doses and compliance with lifestyle recommendations.

## Results

Of the 74 participants enrolled to initiate liraglutide therapy, 55 completed the dose-escalation phase and maintained continuous use of liraglutide for at least six months and were included in the efficacy and safety analysis. Baseline characteristics are shown in **Table 1**. Among the patients studied, two children <12 years had autism spectrum disorder. In the group ≥12 years, comorbidities included: one patient with autism spectrum disorder, three with craniopharyngioma, two with Blount’s disease, two with neuropsychomotor developmental delay, one with muscular dystrophy, and one with steroid-resistant nephrotic syndrome (**Supplementary Material**).

**Table 1:** Baseline demographic, anthropometric, and pubertal characteristics of participants, stratified by age group (6–12 years and >12 years).

Age Group	6-12 years (T0)	>12 years (T0)
n.	23	32
Age (years)	10 ± 1.77	14.9 ± 1.97
Sex (M/F)	11/12	18/14
Stage 1 (B1/G1)	10 (43.5%)	2 (6.3%)
Stage 2 (B2/G2)	4 (17.4%)	2 (6.3%)
Stage 3 (B3/G3)	5 (21.7%)	5 (15.6%)
Stage 4 (B4/G4)	4 (17.4%)	5 (15.6%)
Stage 5 (B5/G5)	0 (0%)	18 (56.2%)
Weight (kg)	77.82 ± 21.44	108.55 ± 28.95
BMI (kg/m²)	34.57 ± 5.16	40.39 ± 8.36
BMI z-score	+4.28 ± 1.10	+3.89 ± 1.16
Waist-to-Height Ratio (WHtR)	0.7 ± 0.06	0.73 ± 0.11
Height (cm)	148.87 ± 12.91	163.30 ± 11.16
Height z-score	+1.23 ± 1.06	–0.09 ± 1.34

Tanner stages refer to breast development in girls (B) and genital development in boys (G). WHtR = waist-to-height ratio; BMI = body mass index (kg/m_2_). Values are presented as mean ± standard deviation (SD).

A total of 19 participants (25,6%) discontinued treatment during the dose-escalation phase: nine participants lost follow-up, eight demonstrated poor adherence to treatment, one patient with ulcerative colitis developed mild gastric bleeding (**Supplementary Material**) and one was referred for bariatric surgery during the follow-up.

### Anthropometric outcomes

**Figure 1:**
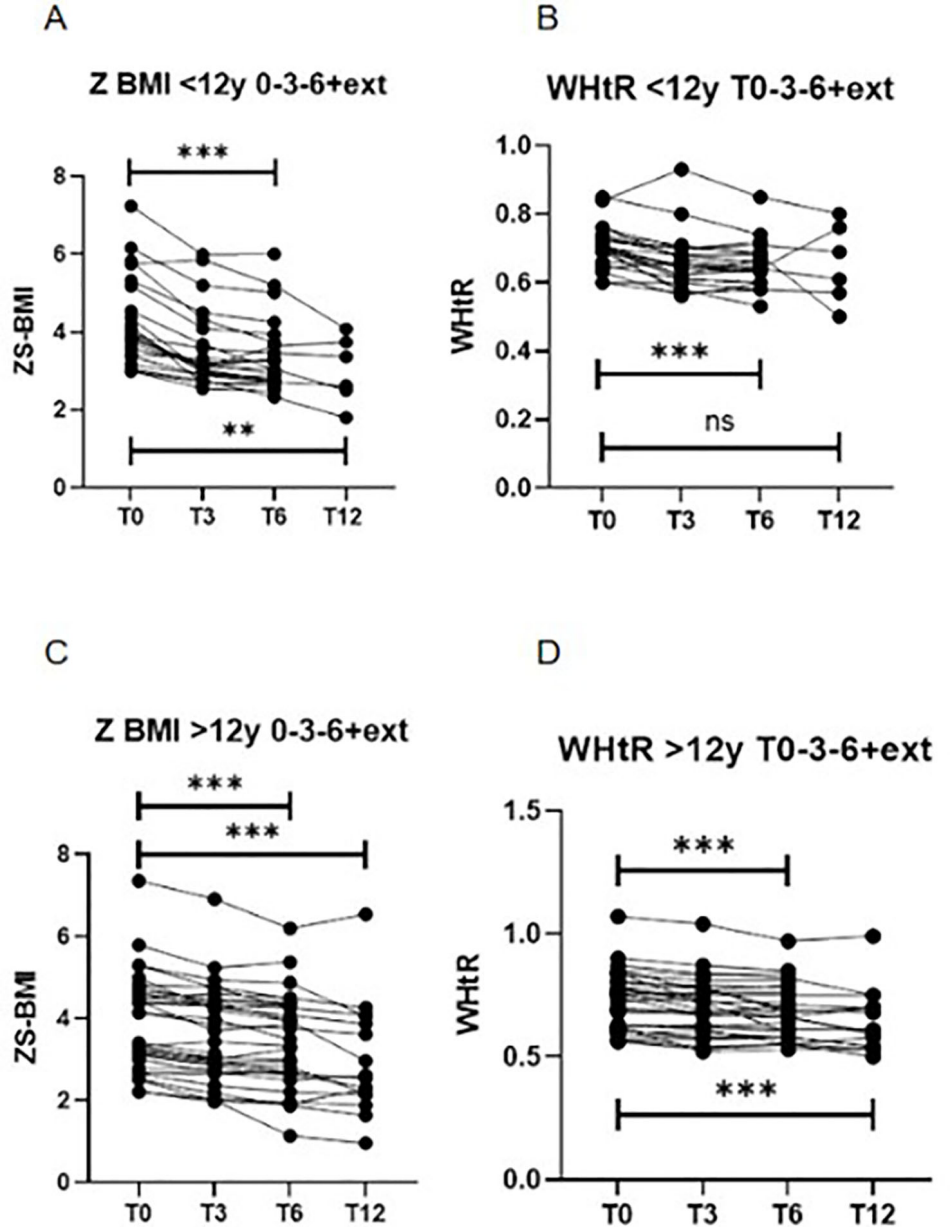
Longitudinal changes in BMI z-score (Z-BMI) and waist-to-height ratio (WHtR) at baseline (T0), 3 months (T3), 6 months (T6), and 12 months (extension, T12), stratified by age group. **(A)** Z-BMI in participants aged 6–12 years (***p < 0.001, **p < 0.01; Friedman test for T0–T3–T6 and T0–T3–T6–T12). **(B)** WHtR in participants aged 6–12 years (***p < 0.001; not significant [ns] for T0–T3–T6–T12; Friedman test). **(C)** Z-BMI in participants aged >12 years (***p < 0.001; Friedman test for T0–T3–T6 and T0–T3–T6–T12). **(D)** WHtR in participants aged >12 years (***p < 0.001; Friedman test for T0–T3–T6 and T0–T3–T6–T12). BMI, body mass index; WHtR, waist-to-height ratio; ns, not significant. .

Significant reductions in BMI (kg/m²), BMI z-score, Weight (kg) and WHtR were observed after six months of treatment. In patients aged 6–12 years, median BMI z-score decreased from +3.90 (3.4 - 5.1) to +3.06 (2.7 - 3.7) (p < 0.0001), and WHtR from 0.70 (0.66 - 0.73) to 0.64 (0.60 - 0.68) (p < 0.0001) (**Table 2**, **Figure 1**). In adolescents aged ≥12 years, BMI z-score declined from +3.77 (3.02 - 4.66) to +3.48 (2.64 - 4.34) (p < 0.0001), and WHtR from 0.74 (0.62 - 0.80) to 0.67 (0.58 - 0.76) (p < 0.0001) (**Table 2**, **Figure 1**). Due to the non-normal distribution of the data, a non-parametric statistical approach was selected.

**Table 2:** Longitudinal changes in anthropometric parameters from baseline (T0) to 3 months (T3) and 6 months (T6), stratified by age group (6–12 years and ≥12 years).

Anthropometric Parameters	T0	T3	T6	*p-*value
6-12 years (n: 23)	Median (p25-p75)	Median (p25-p75)	Median (p25-p75)	
Weight (kg)	74.8 (65.8-88.6)	69.2 (61.8-87.2)	70.0 (61.1-85.4)	<0.0001
BMI (kg/m2)	33.3 (30.7-38.0)	30.8 (27.3-33.9)	30.9 (26.4-33.4)	<0.0001
BMI (ZS)	+3.90 (3.4-5.1)	+3.11 (2.9-4.1)	+3.06 (2.7-3.7)	<0.0001
WHtR	0.70 (0.66-0.73)	0.65 (0.61-0.70)	0.64 (0.60-0.68)	<0.0001
Stature (cm)	149.5 (141-159)	152.0 (143-160.5)	154.0 (144.5-160.5)	<0.0001
Stature (ZS)	+1.39 (0.50-2.03)	+1.31 (0.46-2.04)	+1.17 (0.32-1.88)	≤ 0.05
≥12years (n: 32)				
Weight (kg)	105.6 (86.3-122.3)	103.1 (83.7-119.6)	103.9 (82.9-113.9)	<0.0001
BMI (kg/m2)	38.85 (33.3-46.0)	37.65 (32.9-45.4)	37.6 (32.2-44.3)	<0.0001
BMI (ZS)	+3.77 (3.02-4.66)	+3.43 (2.75-4.40)	+3.48 (2.64-4.34)	<0.0001
WHtR	0.74 (0.62-0.80)	0.69 (0.61-0.77)	0.67 (0.58-0.76)	<0.0001
Stature (cm)	165.5 (154.6-172.8)	165.5 (154.8-174.1)	166.5 (156.3-175.6)	<0.0001
Stature (ZS)	-0.19 ± (1.20-0.95)	-0.16 (1.17-0.69)	-0.19 (1.13-0.69)	<0.0001

Data are expressed as median (25th–75th percentile). *p*-values refer to the comparison between baseline (T0), (T3) and 6 months (T6) using the Friedman test. Statistically significant differences were considered at p < 0.05. BMI = body mass index; ZS = z-score; WHtR = waist-to-height ratio.

**Figure 2:**
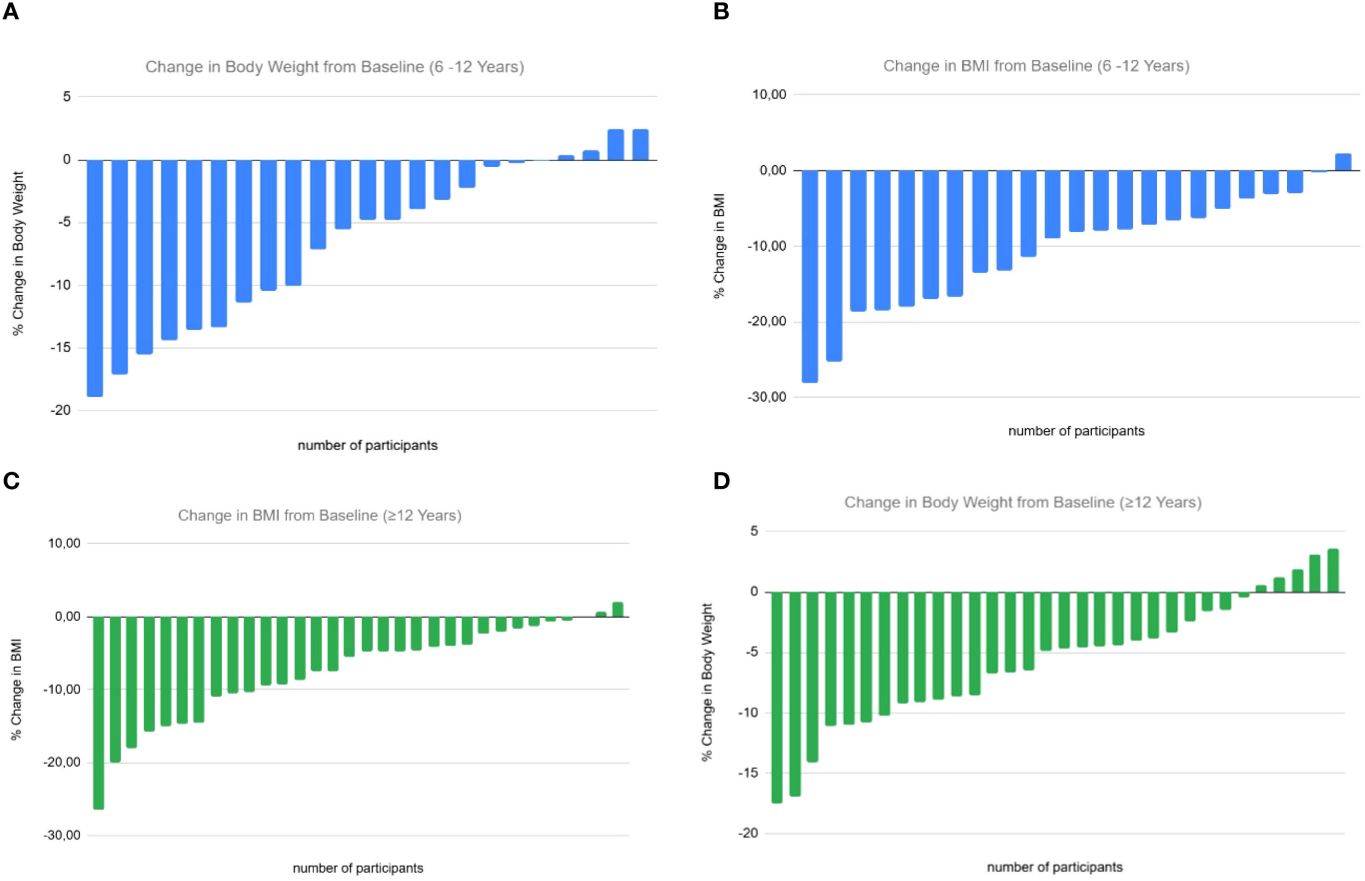
Individual percentage changes in body weight and body mass index (BMI) from baseline following liraglutide treatment, stratified by age group. The X-axis represents individual study participants, and the Y-axis represents the percentage change from baseline. Each bar represents one participant’s change from baseline. **(A)** Percentage change in body weight (kg) in children aged 6–12 years. **(B)** Percentage change in BMI (kg/m²) in children aged 6–12 years. **(C)** Percentage change in BMI (kg/m²) in adolescents aged ≥12 years. **(D)** Percentage change in body weight (kg) in adolescents aged ≥12 years.

In the 6–12 year group, 82.6% experienced a BMI reduction >5%, and 47.8% achieved a reduction >10% after 6 months. Among adolescents ≥12 years, 56.5% had a BMI reduction >5% and 43.5% achieved >10% (**Figure 2**).

In adolescents ≥12 years, BMI z-score declined from +3.77 (3.02–4.66) to +3.48 (2.64–4.34) (*p*<0.0001), and WHtR from 0.74 (0.62–0.80) to 0.67 (0.58–0.76) (*p*<0.0001).

Twenty-two patients (6 aged <12 years) completed 12 months of treatment, with additional reductions in BMI z-score compared to 6 months (6–12 year group: from +3.90 to +2.99, *p* = 0.0028; ≥12 years: from +3.77 to +2.57, *p*<0.0001). Across all participants, liraglutide was associated with a mean weight loss of –5.99 kg (–6.14% of baseline weight) and a mean BMI reduction of –3.35 kg/m² (–8.85%) (**Figure 2**). 

### Metabolic parameters

**Figure 3:**
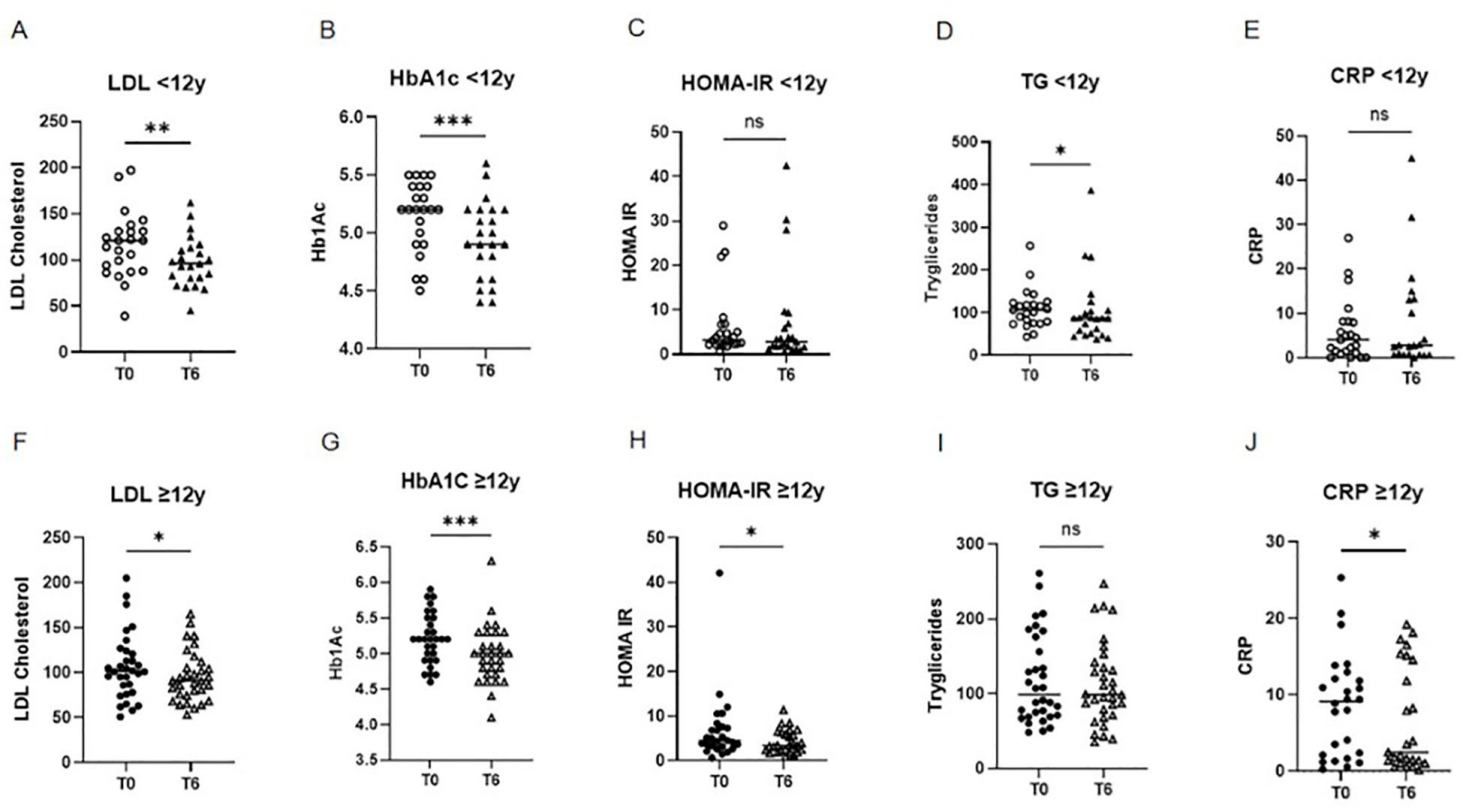
Comparison of metabolic parameters at baseline (T0) and after 6 months (T6) of liraglutide treatment, stratified by age group. Children aged 6–12 years: **(A)** LDL cholesterol (**p < 0.01; paired t-test), **(B)** HbA1c (***p < 0.001; Wilcoxon test), **(C)** HOMA-IR (NS; Wilcoxon test), **(D)** triglycerides (*p < 0.05; Wilcoxon test), **(E)** CRP (NS; Wilcoxon test) Adolescents aged ≥12 years: **(F)** LDL cholesterol (*p < 0.05; paired t-test), **(G)** HbA1c (****p < 0.0001; Wilcoxon test), **(H)** HOMA-IR (**p < 0.05; Wilcoxon test), **(H)** triglycerides (NS; Wilcoxon test), **(E)** CRP (NS; Wilcoxon test) LDL, low-density lipoprotein; HbA1c, glycated hemoglobin; HOMA-IR, homeostatic model assessment of insulin resistance; CRP, C-reactive protein; NS, not significant.

Improvements were observed in several metabolic outcomes. In the 6–12 year group, LDL cholesterol and triglycerides decreased significantly (p<0.01 and <0.05), along with reductions in HbA1c (p<0.001), while HOMA-IR and CRP showed no significant change (**Figure 3**). In the ≥12 year group, LDL cholesterol (*p*<0.05), HbA1c (*p*<0.001), HOMA-IR (*p*<0.05), and CRP (*p*<0.05) decreased significantly, with no significant change in triglycerides (**Figure 3**). No significant changes were observed in HDL cholesterol or uric acid in either group. Due to the non-normal distribution of the data, a non-parametric Wilcoxon test was applied to all metabolic variables, except LDL and HDL cholesterol levels, which were normally distributed and analyzed using the t-test.

### Safety and tolerability

Among the 55 participants, 24 (43.6%) reported mild adverse events, predominantly gastrointestinal, most commonly occurring during the dose-escalation phase. Symptoms included nausea (32.7%), vomiting (16.3%), diarrhea (10.9%), abdominal pain (7.2%), headache (3.6%), belching (1.8%), and drowsiness (1.8%). Symptomatic management, slower dose titration (0.6 mg every 14 days) and dose adjustments were effective in most cases. No serious adverse events were reported, and no abnormalities in liver function tests were detected (**Supplementary Figure 1**). Growth in height was observed across all participants during the study period (**Table 2**).

### Adherence

Fifteen patients (27%) reported inconsistent medication use (>2 missed doses/week). Nutritional counseling adherence was considered satisfactory in 40% of patients, and physical activity guidance was followed adequately by 31%.

## Discussion

This real-world cohort study demonstrated that liraglutide (up to 3.0 mg/day) was associated with reductions in BMI, BMI z-score (Z-BMI), body weight, and waist-to-height ratio (WHtR) among children and adolescents with severe obesity treated in a public quaternary care center in Brazil. Clinically meaningful weight reduction was observed after six months in both age groups, with benefits sustained among those who continued treatment for up to 12 months.

In children under 12 years, Z-BMI decreased by approximately 0.29, BMI by 2.5 kg/m², mean body weight by 4.8 kg, and WHtR by 0.06. The proportion achieving ≥5% and ≥10% BMI reduction was 82.6% and 47.8%, respectively. These results exceed those reported in the SCALE Kids trial, in which 46% of children aged 6 to 12 years achieved a ≥5% reduction in BMI and the mean BMI Z score decreased by –0.7 after 56 weeks of liraglutide treatment (18). Despite the shorter intervention period in our study, the findings suggest that liraglutide may provide clinically meaningful benefits even in the early stages of treatment, supporting its potential use in younger children with severe obesity—despite current regulatory restrictions. However, such comparisons should be interpreted cautiously given differences in study design, population characteristics, and follow-up duration.

These results exceed those reported in the SCALE Kids trial, in which 46% of children aged 6 to 12 years achieved a ≥5% reduction in BMI and the mean BMI Z score decreased by –0.7 after 56 weeks of liraglutide treatment (18). Despite the shorter intervention period in our study, the findings suggest that liraglutide may provide clinically meaningful benefits even in the early stages of treatment, supporting its potential use in younger children with severe obesity—despite current regulatory restrictions. Nevertheless, such comparisons should be interpreted with caution, given the differences in study design, population characteristics, and follow-up duration.

Among adolescents (≥12 years), the mean reduction in Z-BMI was approximately 0.3, absolute BMI decreased by about 1.25 kg/m², and body weight by 1.7 kg over six months. A ≥5% BMI reduction was achieved by 73.9% of participants, and 43.5% achieved ≥10%. These results are greater than those reported in the RCT by Kelly et al. (43.3% ≥5% BMI reduction after 56 weeks), but again, direct comparisons are limited by methodological differences (4). Nonetheless, our findings suggest that liraglutide can induce early and clinically relevant anthropometric changes in adolescents with severe obesity in a real-world setting.

Metabolic improvements included significant reductions in LDL cholesterol and HbA1c in both age groups, while adolescents also showed decreases in HOMA-IR and CRP. The CRP reduction in older patients but not in younger children may reflect greater baseline inflammation or differences in pubertal stage, body composition, or comorbidity profiles. These findings are consistent with the anti-inflammatory and insulin-sensitizing properties of GLP - 1 receptor agonists reported in prior pediatric trials and summarized in the updated Cochrane review by Torbahn et al. (13, 14). However, the absence of consistent changes across all markers suggests that metabolic benefits may vary according to age, baseline risk profile, and treatment duration.

The safety profile of liraglutide in our cohort was consistent with previous pediatric randomized controlled trials (RCTs), with adverse events (AEs) predominantly mild and gastrointestinal in nature, including nausea, vomiting, and diarrhea, reported in 43.6% of participants (4, 18–21). No detrimental effects on linear growth, liver function, or pancreatic enzymes were observed, aligning with prior evidence that liraglutide is generally well tolerated in children and adolescents (4, 18–21).

Despite these favorable outcomes, the discontinuation rate during the dose-escalation phase was relatively high (25.6%), mirroring patterns observed in other pediatric liraglutide trials where treatment withdrawal often occurred during the initial weeks, coinciding with peak gastrointestinal symptoms (18–21). In our setting, most discontinuations were attributed to loss to follow-up or poor adherence, reflecting systemic challenges in public healthcare delivery. A substantial proportion of families were socioeconomically vulnerable, living in remote or underserved areas, where transportation costs, time away from work, and the demands of monthly clinic visits posed significant barriers to sustained engagement. These structural challenges highlight the need for accessible, family-centered care models tailored to low-resource contexts, to ensure equitable implementation of GLP - 1 RA therapy in pediatric obesity.

A key strength of this study lies in its conduct within a publicly funded healthcare system, entirely independent of pharmaceutical industry sponsorship, thereby enhancing both methodological rigor and external validity. The evaluation of treatment adherence in a real-world setting provides valuable translational insight, while the inclusion of a socioeconomically diverse cohort reflects the heterogeneity encountered in routine clinical practice. These characteristics confer greater applicability of our findings to real-world populations and underscore liraglutide’s potential as a pragmatic therapeutic option to reduce health disparities in pediatric obesity, particularly in underserved communities (22, 23).

While adherence to liraglutide therapy was acceptable, compliance with lifestyle recommendations, especially regarding physical activity, remained suboptimal. This gap emphasizes the critical need for integrated, multidisciplinary, and sustainable care strategies to support long-term behavioral change (23–26).

The presence of complex comorbidities in a subset of participants—such as syndromic or hypothalamic obesity, neurodevelopmental disorders, and chronic systemic diseases—may influence therapeutic responsiveness, potentially attenuating the magnitude of treatment effects. Although these conditions were not exclusion criteria, they illustrate the considerable clinical heterogeneity of pediatric obesity in real-world practice and highlight the need for individualized, multidisciplinary treatment strategies. The absence of stratified analyses by these subgroups in the present study represents a limitation, as such analyses could provide valuable insight into subgroup-specific treatment efficacy.

Importantly, the primary therapeutic goal in managing pediatric obesity extends beyond absolute weight reduction to encompass the mitigation of long-term cardiometabolic risk. As a chronic, multifactorial disease, obesity warrants the same clinical rigor in diagnosis, monitoring, and treatment as other chronic conditions such as dyslipidemia or type 2 diabetes mellitus (9, 17). Reframing obesity as a chronic disease—rather than attributing it to individual behavioral failure—may help reduce weight-related stigma and improve patient and family engagement in evidence-based care (24, 25).

Our findings support the consideration of pharmacological therapy as a component of pediatric obesity management when lifestyle interventions alone fail to achieve clinically meaningful outcomes. However, this recommendation should be interpreted with caution, as variability in treatment responsiveness, particularly among younger children and those with complex comorbidities, may limit the generalizability of results. Persistent reluctance to initiate anti-obesity pharmacotherapy in pediatric populations remains a barrier to timely intervention. Nonetheless, existing evidence suggests that adverse effects of these therapies are generally mild and reversible, whereas the long-term health consequences of untreated obesity are substantial (6, 7, 17). Therefore, early intervention—utilizing the most effective and evidence-based options available—should be considered within the context of a comprehensive care model, with shared responsibility among families, clinicians, and policymakers to ensure equitable and timely access to obesity treatment.

Several limitations warrant consideration. The absence of a control group limits causal inference, and the modest sample size reduces the precision of estimates. Anthropometric assessment relied solely on waist-to-height ratio (WHtR) and BMI, without incorporating more precise measures of body composition such as dual-energy X-ray absorptiometry (DXA) or bioelectrical impedance analysis (BIA). Additionally, potential confounding by unmeasured factors, including dietary patterns and psychosocial variables, cannot be excluded. In summary, this study suggests that liraglutide may have a role as part of a comprehensive management strategy for severe pediatric obesity, including in younger children, within publicly funded healthcare systems. The observed anthropometric and metabolic improvements are encouraging, yet the small sample size, and lack of a control group limit the strength of these findings and preclude definitive recommendations for routine clinical use. High attrition and variability in lifestyle adherence further underscore the need for both prospective, adequately powered randomized trials and well-designed real-world studies in diverse and socioeconomically heterogeneous populations to confirm long-term efficacy, safety, cost-effectiveness, and feasibility in resource-limited settings.

## Data Availability

The original contributions presented in the study are included in the article/**Supplementary Material**. Further inquiries can be directed to the corresponding author.
