# Supplementary material for: 12-month outcomes of GLP - 1 in severe pediatric obesity: real-world data
Source: Front Endocrinol (Lausanne). 2025 Sep 19;16:1663499. doi: 10.3389/fendo.2025.1663499 (PMC12491053; doi:10.3389/fendo.2025.1663499)
Supplement: Supplementary file 1 [file DataSheet1.docx]

**SUPPLEMENTARY MATERIAL**

| **Comorbidities and characteristics of Excluded Patient** | |
| --- | --- |
| **Ulcerative colitis** | **A.G.M.B., male, 17 years old**  The patient had a diagnosis of ulcerative colitis since the age of 7. At the time of inclusion in the treatment protocol, the disease was in clinical remission under maintenance therapy with mesalazine. On the third day of liraglutide treatment (0.6 mg/day), he presented with an episode of hematochezia, prompting immediate discontinuation of the medication and referral for further evaluation by pediatric gastroenterology. No evidence of disease relapse was identified. The patient was later submitted to bariatric surgery, which resulted in a 20% reduction in preoperative body weight. |

| **Comorbidities and characteristics of Included Patient** | |
| --- | --- |
| **Nephrotic syndrome** | **D.A.F., male, 13 years old**  The patient had a history of steroid-dependent nephrotic syndrome, with the first episode of decompensation occurring at 2 years of age, and underwent multiple courses of corticosteroid therapy throughout childhood. At the time of study enrollment, he was receiving low-dose prednisolone (2.5 mg/day), with a tapering schedule already in place. He remained off corticosteroids until the 7th month of liraglutide treatment, when he experienced a relapse of nephrotic syndrome, evidenced by laboratory-confirmed proteinuria, but without associated edema or weight gain. High-dose corticosteroid therapy (prednisolone 60 mg/day) was reinitiated, followed by gradual tapering and complete discontinuation by the 11th month of liraglutide treatment. As this was a real-world observational study, the patient remained classified within the >12-year-old age group. The patient completed 12 months of treatment with liraglutide with an initial BMI of 48.9 kg/m² and a final BMI of 41.2 kg/m², representing a loss of 15.75% of the initial BMI. |


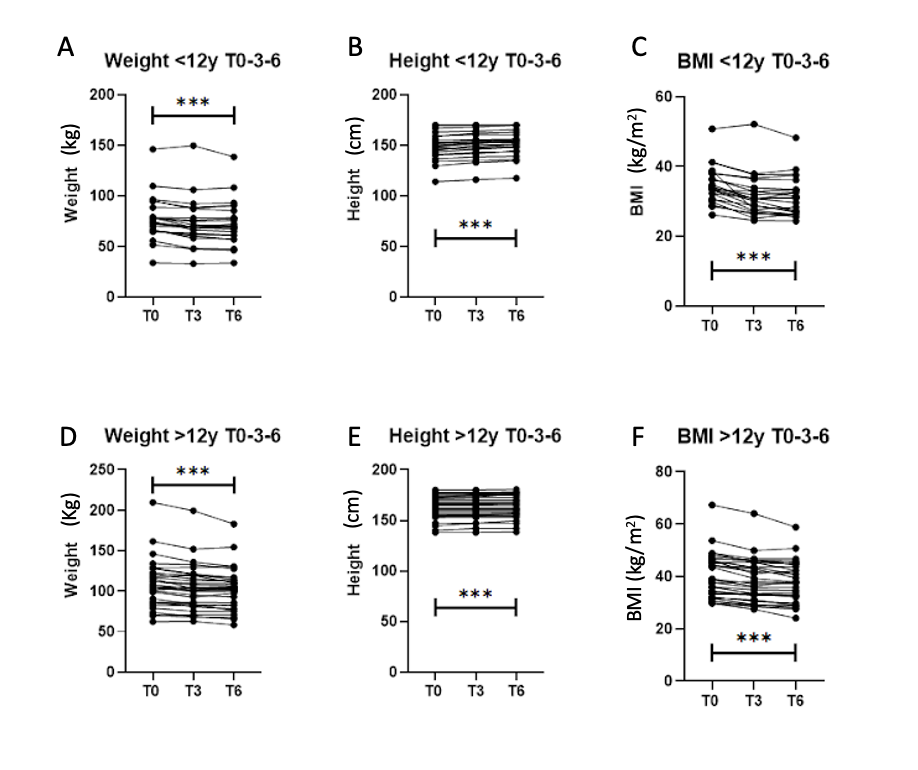


**Supplementary figure 1: Weight, Height and BMI according to age group at Baseline (T0) , 3 months (T3) and 6 months (T6).** A. Weight Patients aged 6 to 12 years (***Friedman T0-3-6,. T0= baseline, T3= 3 months, T6= 6 months). B. Height -Patients aged 6 to 12 years (***Friedman - T0-3-6) C. BMI-Patients Patients aged 6 to 12 years (*** Friedman - T0-3-6,*** Friedman -T0-3-6)  D. Weight Patients aged >12 years (***Friedman T0-3-6). E. Heigh >12 yearst -Patients aged 6 to 12 years (***Friedman - T0-3-6) F. BMI-Patients Patients aged>12 years (*** Friedman - T0-3-6,*** Friedman -T0-3-6)

|  | | | |
| --- | --- | --- | --- |
| ***Metabolic and safety parameters*** | **T0** | **T6** | ***p-*value** |
| ***6-12 years*** |  |  |  |
| Cholesterol |  |  |  |
| Total | 168.7 ± 33.31 | 163.4 ± 34.80 | 0.24 |
| HDLc | 43.83 ± 8.02 | 42.76 ± 8.30 | 0.89 |
| LDLc | 116.4 ± 35.59 | 97.96 ± 27.31 | 0.002 |
| Triglycerides | 108.7 ± 46.30 | 104.8 ± 80.63 | 0.046 |
| Fasting plasma glucose (mg/dL) | 81.43 ± 7.15 | 80.92 ± 6.05 | 0.55 |
| HBA1c | 5.14 ± 0.30 | 4.93 ± 0.33 | <0.001 |
| HOMA-IR | 6.32 ± 7.57 | 7.24 ± 10.99 | 0.51 |
| CRP | 5.95 ± 6.94 | 8.08 ± 10.66 | 0.56 |
| Uric Acid | 4.81 ± 1.16 | 4.31 ± 1.55 | 0.32 |
| AST | 21.66± 5.91 | 20.85± 5.29 | 0.57 |
| ALT | 21.53± 8.07 | 17.04± 5.73 | 0.01 |
| ***>12 years*** |  |  |  |
| Cholesterol |  |  |  |
| Total | 161.6 ± 39.40 | 153.5 ± 31.42 | 0.31 |
| HDLc | 41.26 ± 7.44 | 40.91 ± 7.17 | 0.80 |
| LDLc | 106.6 ± 36.52 | 95.70 ± 28.35 | 0.03 |
| Triglycerides | 118.4 ± 59.59 | 112.6 ± 55.10 | 0.79 |
| Fasting plasma glucose (mg/dL) | 82.09 ± 9.43 | 77.89 ± 7.69 | 0.06 |
| HBA1c | 5.21 ± 0.35 | 4.98 ± 0.41 | <0.001 |
| HOMA-IR | 6.83 ± 7.84 | 4.26 ± 2.60 | 0.01 |
| CRP | 8.60 ± 6.70 | 6.70 ± 6.90 | 0.02 |
| Uric Acid | 5.46 ± 1.26 | 5.28 ± 1.27 | 0.77 |
| AST | 21.66± 5.91 | 20.85± 5.29 | 0.57 |
| ALT | 30.46± 17.59 | 29.78± 21.83 | 0.39 |

**Supplementary Table 1. Baseline (T0) and 6-Month (T6) Metabolic and Safety Data Stratified by Age Group (6-12 years and ≥ 12 Years)** Legend: Values are expressed as mean ± standard deviation. *p*-values refer to the comparison between baseline (T0) and 6 months (T6) using paired analysis (Wilcoxon test). Statistically significant differences were considered at *p* < 0.05. Abbreviations: BMI, body mass index; ZS, z-score; WHtR, waist-to-height ratio; HDLc, high-density lipoprotein cholesterol; LDLc, low-density lipoprotein cholesterol; HBA1c, glycated hemoglobin; HOMA-IR, homeostasis model assessment for insulin resistance; CRP, C-reactive protein; AST, aspartate aminotransferase test; ALT,alanine aminotransferase test.
